# Determinant of m^6^A regional preference by transcriptional dynamics

**DOI:** 10.1093/nar/gkae169

**Published:** 2024-03-07

**Authors:** Yalan Wang, Shen Wang, Zhen Meng, Xiao-Min Liu, Yuanhui Mao

**Affiliations:** Department of Neurology of The Second Affiliated Hospital & Liangzhu Laboratory, Zhejiang University School of Medicine, Hangzhou, China; Shanghai Key Laboratory of Metabolic Remodeling and Health, Institute of Metabolism and Integrative Biology, Institute of Reproduction and Development, Fudan University, Shanghai, China; School of Life Science and Technology, China Pharmaceutical University, Nanjing, China; Department of Neurology of The Second Affiliated Hospital & Liangzhu Laboratory, Zhejiang University School of Medicine, Hangzhou, China; School of Life Science and Technology, China Pharmaceutical University, Nanjing, China; Department of Neurology of The Second Affiliated Hospital & Liangzhu Laboratory, Zhejiang University School of Medicine, Hangzhou, China

## Abstract

*N^6^*-Methyladenosine (m^6^A) is the most abundant chemical modification occurring on eukaryotic mRNAs, and has been reported to be involved in almost all stages of mRNA metabolism. The distribution of m^6^A sites is notably asymmetric along mRNAs, with a strong preference toward the 3′ terminus of the transcript. How m^6^A regional preference is shaped remains incompletely understood. In this study, by performing m^6^A-seq on chromatin-associated RNAs, we found that m^6^A regional preference arises during transcription. Nucleosome occupancy is remarkedly increased in the region downstream of m^6^A sites, suggesting an intricate interplay between m^6^A methylation and nucleosome-mediated transcriptional dynamics. Notably, we found a remarkable slowdown of Pol-II movement around m^6^A sites. In addition, inhibiting Pol-II movement increases nearby m^6^A methylation levels. By analyzing massively parallel assays for m^6^A, we found that RNA secondary structures inhibit m^6^A methylation. Remarkably, the m^6^A sites associated with Pol-II pausing tend to be embedded within RNA secondary structures. These results suggest that Pol-II pausing could affect the accessibility of m^6^A motifs to the methyltransferase complex and subsequent m^6^A methylation by mediating RNA secondary structure. Overall, our study reveals a crucial role of transcriptional dynamics in the formation of m^6^A regional preference.

## Graphical Abstract

**Graphical Abstract:**
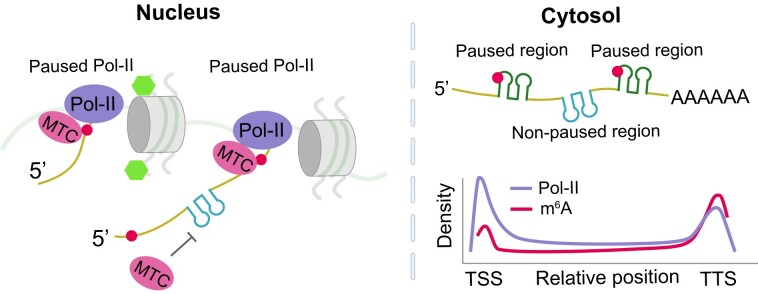

## Introduction

RNA modifications are emerging players in both transcriptional and post-transcriptional regulation (1,2). To date, more than 150 distinct RNA modifications have been identified. Among them, *N^6^*-methyladenosine (m^6^A) is the most abundant internal base modification found in eukaryotic messenger RNAs (mRNAs) (3,4). m^6^A exhibits considerable dynamics across tissues and species (5), which is critical for its diverse functions in RNA metabolism. Regardless of the context-dependent methylation among different mRNAs, m^6^A sites within transcripts generally display consistent tendencies across most mRNA species. Notably, m^6^A is not uniformly distributed along mRNAs, with a remarkable increase in the 3′ untranslated region (UTR) of the mRNA. In addition, m^6^A methylation sites are enriched around splicing sites (6) and polyadenylation sites (7). Increasing evidence suggests that m^6^A sites on mRNAs can serve as road markers to recruit various regulatory factors (8–10). Therefore, understanding the mechanisms driving regional preference for m^6^A sites is critical to understanding of m^6^A-mediated gene expression regulation.

Most m^6^A sites are believed to be co-transcriptionally installed by the methyltransferase complex (MTC), which specifically recognizes the m^6^A motif RRACH (11–13). During transcription, numerous factors have been identified to facilitate the binding of MTC to the nascent transcript and subsequent m^6^A installation. For example, the DNA binding protein CEBPZ can recruit METTL3 (14), a m^6^A writer, to the transcript start site (TSS) of actively transcribed genes. The CEBPZ-guided recruitment of METTL3 subsequently promotes m^6^A modification within the coding region of associated mRNAs. In addition, another study using a computation model reported that slowdown of early transcription elongation increases the recruitment of MTC to TSS (15). However, the mechanism explaining the relationship between MTC binding to TSS and downstream m^6^A methylation on mRNA remains unclear. Intriguingly, a previous study reported an association between m^6^A modification and histone H3 trimethylation at lysine 36 (H3K36me3) (16). H3K36me3 is directly recognized by METTL14, facilitating the binding of MTC to adjacent RNA polymerase II, and promoting m^6^A modification of actively transcribed RNAs. While H3K36me3 tends to be enriched in the gene body and the 3′ end of the gene, mimicking the distribution of m^6^A sites, the fact that the number of H3K36me3 sites is tenfold higher than that of m^6^A sites suggests the involvement of other factors in determining methylation sites. Indeed, several recent studies have revealed that the exon junction complex (EJC), a complex deposited 24 nucleotides upstream of splicing junctions, can act as a m^6^A suppressor, protecting mRNA regions from methylation (17–19), and thus influencing the m^6^A landscape along transcripts. However, a later study (20) reported notable diversity of m^6^A methylation levels in ECJ free regions, implying additional factors or elements contributing to local m^6^A methylation.

RNA transcription is an intrinsically dynamic process, involving frequent interactions between the elongating complex, nucleosomes, histone markers, and RNA structures. Previous studies have revealed a crucial role of local m^6^A methylation in transcription dynamics, such as promoting R-loop formation (9,21) and affecting RNA splicing (22). However, it remains unclear whether transcription dynamics influence m^6^A methylation. A previous study found that either transcription initiation or elongation rate has a significant effect on global m^6^A methylation levels (23). It is interesting that treatment with the transcription inhibitor Camptothecin (CPT) can increase m^6^A methylation levels on total mRNAs (23). However, neither the mRNAs nor the regions of mRNAs, where the m^6^A methylation is affected upon transcription inhibition, have been clearly defined in previous studies. In the current study, we performed a comprehensive analysis of co-transcriptional regulation contributing to m^6^A regional preference by integrating various high-throughput sequencing datasets. Our analyses reveal a dramatic enrichment of m^6^A methylation in the regions where the transcription elongation rate is slow. Our results suggest a rate-dependent m^6^A methylation contributing to m^6^A regional preference along the transcript.

## Materials and methods

### Cell culture and transcription elongation inhibitor treatment

MEF cells were grown in DMEM (BI) medium containing 10% FBS and 1% penicillin–streptomycin. Mouse embryonic stem cells J1 were cultured in KO-DMEM (Thermo Fisher) base medium supplemented with 1 mM glutamax (Invitrogen), 1% non-essential amino acids (Invitrogen), 15% KOSR (Thermo Fisher), 1000 U ml-1 LIF (Millipore), and 100 μM β-ME (Sigma) on plates coated with 0.2% gelatin. For flavopiridol treatment assays, MEF cells at around 80% confluence were replenished with fresh medium 2 h before harvesting. Subsequently, the cells were treated with 300 nM flavopiridol for a duration of 30 min.

### Cellular fractionation and chrRNA isolation

Cells were washed with chilled PBS and pelleted by centrifugation before they were subjected to cell membrane lysis buffer (0.15% NP-40, 10 mM Tris–HCl pH 7.0, 150 mM NaCl, 25 μM α-amanitin, 10 U SUPERase_In) and incubated on ice for 5 min. Cell lysates were then added to 2.5 volumes of chilled sucrose cushion (10 mM Tris–HCl pH 7.0, 150 mM NaCl, 25% sucrose, 25 μM α-amanitin, 20 U SUPERase_In) and the mixture was centrifuged at 4°C with 16 000 × g for 10 min. The supernatant was the cytoplasmic fraction, and the pellet was regarded as cell nuclei. The nuclear pellet was then washed with nuclear wash buffer (0.1% Triton X-100, 1 mM EDTA, in 1 × PBS, 25 μM α-amanitin, 10 U SUPERase In, and protease inhibitor mix) twice and pelleted by centrifugation at 1150 × g for 2 min. The supernatant was discarded, and the pellet was subjected to chilled glycerol buffer (20 mM Tris–HCl pH 8.0, 75 mM NaCl, 0.5 mM EDTA, 50% glycerol, 0.85 mM DTT, 25 μM α-amanitin, 10 U SUPERase_In). Equal volumes of chilled nuclei lysis buffer (1% NP-40, 20 mM HEPES pH 7.5, 300 mM NaCl, 1 M urea, 0.2 mM EDTA, 1 mM DTT, 25 μM α-amanitin, 10 U SUPERase_In) were then added to the nuclei suspension and incubated on ice for 2 min followed by centrifugation at 4°C at 18 500 × g for 2 min. The pellet was subjected to chilled chromatin resuspension solution (25 μM α-amanitin, 20 units SUPERase_In, 1 × protease inhibitor mix (in 1 × PBS)) to obtain the insoluble chromatin-associated fraction and subjected to RNA extraction using TRIzol Reagent.

### m^6^A dot blot assay

For m^6^A dot blots, equal amounts of chrRNA samples were spotted on the Amersham Hybond-N+ membrane and crosslinked with UV at 254 nm. The membrane was blocked with PBST containing 5% non-fat milk and 0.1% Tween-20 for 1 h and incubated with anti-m6A antibody (Abcam) overnight at 4°C. The membrane was then incubated with HRP-conjugated anti-rabbit IgG (1:5000 dilution) at room temperature for 1 h and visualized by using enhanced chemiluminescence (Tanon). Following exposure, the membrane was incubated with methylene blue (Sigma) for 15 min, and then washed with nuclease-free water briefly before capture.

### m^6^A immunoprecipitation and high throughput sequencing

For m^6^A immunoprecipitation, 50 μg chrRNA samples were fragmented by RNA fragmentation buffer (100 mM Tris–HCl pH 7.0 and 100 mM ZnCl_2_) at 94°C for 30 s. Flowing standard ethanol precipitation and 1/20 of the fragmented RNA was saved as the input. The remaining fragmented RNA was immunoprecipitated with anti-m^6^A antibody (Abcam) in IP buffer (0.1% NP-40, 10 mM Tris–HCl pH 7.0, 150 mM NaCl) at 4°C for 4 h. Dynabeads protein A/G (NEB) were washed, added to the mixture and incubated for 2 h at 4°C with rotation. The m^6^A-containing RNA fragment was eluted twice with 6.7 mM N6-methyladenosine 5′-monophosphate sodium salt at 4°C for 1 h and precipitated with ethanol at –80°C overnight. RNA was dissolved in 10 μl nuclease-free water and subjected to reverse transcription for qPCR (see reverse transcription and quantitative Real Time-PCR). For chrRNA m6A-seq, cDNA library construction and sequencing were performed at LC-BIO Biotech Ltd. (Hangzhou, China). Paired-end 2 × 150 bp sequencing on an Illumina NovaSeq™ 6000 platform was also performed on libraries.

### Reverse transcription and quantitative real time-PCR

m^6^A immunoprecipitation was performed according to the procedure described above. Immunoprecipitated m^6^A RNAs and input RNAs were reverse transcribed into cDNA using a HiScript III 1st Strand cDNA Synthesis Kit plus gDNA wiper (Vazyme) following the manufacturer's instructions. qPCR was carried out using ChamQ SYBR qPCR Master Mix (Vazyme) and monitored by a QuantStudio three Real-Time PCR System (Applied Biosystems). The primers used for qPCR were designed based on the positions of m^6^A sites. The list of primers is as follows (from 5′ to 3′; qF stands for forward primer; qR stands for reverse primer):

Gnai2-qF (140), GGAACTGCGGACCTGAGAG;

Gnai2-qR (140), CTTGTCCTCGGCGCTCAC;

Gnai2-qF (19702), CAAGGAGATCTACACGCACT;

Gnai2-qR (19702), TCAGAAGAGGCCACAGTCC;

Cenpu-qF (64), CTGTTCAGAGCAAGACCGG;

Cenpu-qR (64), TCTCCTTGCAGCCATCCTAC;

Cenpu-qF (10851), CTCCATCCCTAGTCACTCGG;

Cenpu-qR (10851), ATCCACGGACGGCCAGATT.

### Collection of public datasets

ChIP-seq data and MNase-seq data were obtained from the work of Chronis *et al.* (24). ATAC-seq data were obtained from Brumbaugh *et al.* (25). PRO-seq data were obtained from Mahat et al. (26). NET-seq data were obtained from Liu *et al.* (27) and Mayer *et al.* (28). chrRNA and ttlRNA m^6^A-seq were obtained from our previous studies (29,30). Single resolution m^6^A sites on nascent RNAs were downloaded from Ke *et al.* (31). The processed icSHAPE activity data were downloaded from Sun *et al.* (32).

### Reads alignment

Sequencing adaptors and low-quality reads were trimmed by Cutadapt (33). The clean reads were then aligned to the mouse (GRCm38) or human (GRCh38) genome using Bowtie with the parameters -a –best -m1–strata. For paired-end ATAC-seq, the parameter -X 1000 was specified to include the long-inserted fragments. Duplicates of ATAC-seq were removed using Picard. Only the reads that uniquely aligned to the genome were used in the following analysis.

### Nucleosome positioning

We used Danpos2 (34), with the parameters -p 1 -a 1, to call nucleosome positions from MNase-seq and ATAC-seq data. For ATAC-seq data, only fragments with lengths >140 bp were used.

### CHIP-seq peak calling

Histone modification peaks were called using MACS2 (35), with the parameters –nomodel –extsize 150 –keep-dup all –call-summits. The MNase-seq data were used as background controls.

### Analysis of PRO-seq and NET-seq data

The reads that aligned to the reverse strand (i.e. the FLAG is 16 in SAM files) were retained, and the 5′ end of the aligned reads, which corresponds to the 3′ end of the nascent RNA fragment, was recorded. To focus our analysis on Pol-II, only the reads aligned to protein coding genes were used. To calculate Pol-II densities around m6A or unmodified RRACH motifs, a sliding window with 50 nucleotides in length and a step of 10 nucleotides was used. Sequencing reads in each sliding window were counted, and then normalized by the average reads of the corresponding gene. The normalized reads in the windows with the same distance to m6A or RRACH motifs were averaged.

### m^6^A-seq peak calling

For m6A-seq, we identified m^6^A sites using a non-parameter method modified from Schwartz *et al.* (36). In brief, read coverages of each protein coding gene were calculated. Then a sliding window of 50 nucleotides with a step of 25 nucleotides was employed to scan each gene. For each window with maximum read coverage higher than 10, a peak-over-median score (POM) was derived by calculating the ratio of the mean read coverage in the window to a trimmed mean read coverage of the corresponding transcript. The trimmed mean coverage referred to the averaged coverage of the gene after trimming the top 10% and bottom 10% values. The windows with POM higher than 3 in the IP sample were remained. The same processes were performed in input sample. The windows found in the input sample were eliminated from the following analyses. The windows that overlapped at least a single nucleotide were merged into one cluster. Finally, a peak over input (POI) score was assigned to each cluster by calculating the ratio of POM in the IP sample to that in the input sample. Clusters with POI scores higher than 3 were retrieved, and defined as m^6^A-enriched clusters. The peak position with maximum coverage in each m6A-enriched cluster was defined as the position of the m^6^A peak. The adenosine site of the nearest RRAC motif was defined as the m^6^A site.

### Differential expression analysis

First, NET-seq reads around the m^6^A sites (−150 bp to +150 bp) were counted, respectively. Then, DESeq2 (37) was used to identify m^6^A sites with significantly changed Pol-II densities upon METTL3 knockout. The sites with an estimated FDR <0.05 were considered significantly changed sites.

### Motif analysis

Motif analyses were performed by MEME (38).

### Statistical analysis

All values and error bars in graphs are presented as the mean ± s.e.m. Differences between groups were analyzed by the unpaired two-tailed Student's *t* test. *P* < 0.05 was considered statistically significant. *P < 0.05. ***P < 0.001.

## Results

### Co-transcriptional methylation shapes m^6^A regional preference

m^6^A methylation level on a transcript is regulated by the balance of methylation and de-methylation processes. While the majority of m^6^A sites are believed to be deposited during co-transcriptional processes, it has been reported that post-transcriptional de-methylation can occur in specific regions of the transcript under certain circumstance, contributing to m^6^A regional preference (39). To investigate when the general m^6^A regional preference in the transcript is established, we compared the distribution of m^6^A sites on chromatin-associated RNAs (chrRNAs) with that on total RNAs (ttlRNAs), primarily composed of mature RNAs (Supplementary Figure S1A). Previously, we have conducted m^6^A-seq of chrRNAs (29,30), which showed an obvious preference for local m^6^A sites on chrRNAs. This result implied that m^6^A regional preference could be established in a co-transcriptional manner.

**Figure 1.**
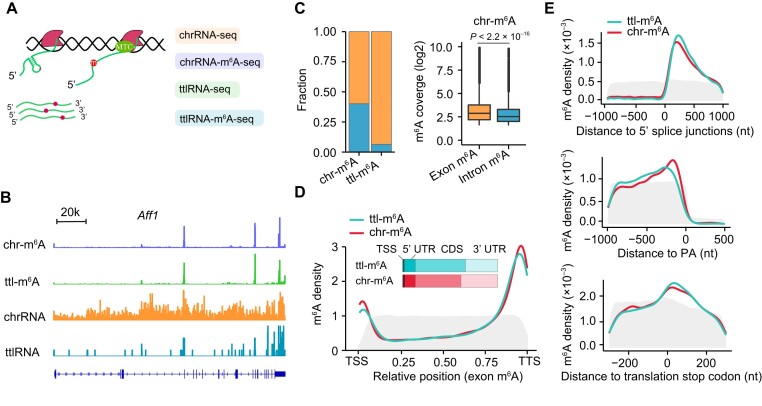
Comparison of m^6^A regional specificity of chrRNAs and ttlRNAs. (**A**) Schematic view of m^6^A-seq and RNA-seq of fractionated RNA species. ttlRNA: total RNA; chrRNA: chromatin-associated RNA. MTC: methyltransferase complex. Pol-II: RNA polymerase II. (**B**) A representative example (*Aff1*) of m^6^A sites in chrRNAs and ttlRNAs. (**C**) A bar plot (left panel) showing the fraction of identified m^6^A sites in exons and introns of chrRNAs and ttlRNAs. The boxplot (right panel) shows peak coverage (normalized by RNA-seq) of intron and exon m^6^A in chrRNAs (Wilcox-test, *P* < 2.2 × 10^–16^). (**D**) Distribution of m^6^A sites along transcripts, showing a similar regional preference of m^6^A sites between chrRNAs and ttlRNAs. RRACH motifs in transcripts are shown as grey frames. Bar plot shows m^6^A sites in different mRNA regions: TSS, 5′ UTR, CDS and 3′UTR. Intron m^6^A sites are not included due to their low frequency in ttlRNAs. (**E**) m^6^A density around the splicing sites (top), polyadenylation signals (middle) and translation stop codon (bottom). To analyze the m^6^A density around splicing sites, only the exons with lengths >500 nt were considered.

To systematically investigate the molecular characteristics of co-transcriptional m^6^A methylation, we re-analyzed our m^6^A-seq of chrRNAs in parallel with m^6^A-seq of ttlRNAs in mouse embryonic fibroblasts cells (MEFs) (Figure 1A). By counting the reads aligned to the mouse genome, chrRNAs showed significantly higher intron reads than ttlRNAs (Figure 1B, Supplementary Figure S1A, Wilcox-test, *P* < 2.2 × 10^–16^), confirming the quality of chrRNA m^6^A-seq. We developed a non-parameter peak calling method (see methods), adapted from Schwartz *et al.* (36), to identify m^6^A peaks. In total, 46 472 m^6^A peaks on chrRNAs (chr-m^6^A) and 11 089 m^6^A peaks on ttlRNAs (ttl-m^6^A) were identified. Consistent with previous reports, there was an obvious enrichment of the motif RRACU near identified m^6^A peaks (Supplementary Figure S1B and S1C). Within the region surrounding the peaks, 34% of the peaks can be assigned to a single consensus motif. Interestingly, m^6^A coverage positively correlates with the number of motifs around the m^6^A peak (Supplementary Figure S1D), suggesting that multiple motifs, if any, within a m^6^A peak region are likely to be methylated. For simplification, the motif nearest to a m^6^A peak is defined as a representative m^6^A site for the peak and used for subsequent analysis.

By comparing m^6^A sites between chrRNAs and ttlRNAs, the majority of ttl-m^6^A sites (∼80%) overlap with chrRNAs (Supplementary Figure S1E). However, the number of chr-m^6^A sites is three-fold higher than ttl-m^6^A sites, implying either the presence of post-demethylation process or that m^6^A containing RNAs are preferentially degraded in the cytosol (40). Notably, the distribution of m^6^A sites on the transcripts remains unchanged regardless of whether the sites are preserved between chrRNAs and ttlRNAs or not (Supplementary Figure S1E), suggesting that the m^6^A regional preference is established during transcription.

To further characterize m^6^A regional preference, we investigated m^6^A sites in specific regions of the transcript. Firstly, we categorized m^6^A sites into introns and exons. As expected, more than 90% of ttl-m^6^A sites were located in exons (exon m^6^A, Figure 1C). In contrast, chr-m^6^A sites were found in both introns and exons (45.8% intron m^6^A). Introns have significantly lower m^6^A modification levels than exons (Figure 1C, Supplementary Figure S1F, Wilcox-test, *P* < 2.2 × 10^–16^), despite the number of consensus motifs in introns being more than tenfold higher than that in exons. The identified intron m^6^A sites are unlikely artifacts because intron m^6^A and exon m^6^A exhibit comparable sensitivity to the silencing of METTL3 (Supplementary Figure S1G).

To gain further insights into the m^6^A regional preference, we next examined the locations of m^6^A sites within chrRNAs and ttlRNAs. While m^6^A consensus motifs were distributed almost uniformly along the transcript, both chrRNAs and ttlRNAs exhibited a strong regional preference for m^6^A locations, with m^6^A enriched in the last and first exons (Figure 1D). The regional preference between chr-m^6^A and ttl-m^6^A remained consistent when segmenting mRNAs into different regions: TSS, 5′ UTR, CDS and 3′UTR. Moreover, in line with previous analyses of m^6^A sites on total RNAs (6,7), our chr-m^6^A and ttl-m^6^A sites were enriched in the regions near the 5′ splicing junctions, polyadenylation signals, and 3′ UTR near the translation stop codon (Figure 1E).

Taken together, we compared m^6^A distribution on chromatin-associated RNAs and total RNAs, which showed a congruent m^6^A regional preference on the whole transcript and local regions including introns/exons, TSS sites, splicing junctions and UTRs of the two types of RNAs, suggesting the co-transcriptional establishment of m^6^A regional preference.

### Nucleosomes are enriched in the downstream region of m^6^A sites

In previous studies, m^6^A has been linked to various histone markers, such as H3K36me3 (16), H3K9me2 (8) and H3K27me3 (41), across different species and context. While it has been proposed that histone markers, such as H3K36me3, can promote local m^6^A methylation in human cells (16), such a correlation is absent at least in fruit fly cells (15), despite the conservation of m^6^A regional preference across species including fruit fly (36,42). Given the substantial dynamics observed in both m^6^A and histone markers across cell context and species, it remains unclear whether the general m^6^A regional preference results from specific distribution of histone markers across the genome. To address this question, we analyzed densities of various histone markers around m^6^A sites using our chrRNAs m^6^A-seq and CHIP-seq datasets from the MEF cells. In agreement with previous studies (16), our analysis in MEF cells revealed an enrichment of H3K36me3 around m^6^A sites (Supplementary Figure S2). Approximately, 35% (16 417 of 46 472 sites) of chr-m^6^A sites in MEF cells overlapped with H3K36me3, which was mainly located toward the 3′ end of the transcript. Intriguingly, in addition to H3K36me3, we also observed an accumulation of various other histone modifications around 80 bp downstream of m^6^A sites (Supplementary Figure S2). Given the distinct characteristics of these histone modifications, it is less likely that m^6^A regulates all these different histone modifications, or vice versa. Instead, the enrichment of different histone modifications implies an association of m^6^A modification with downstream nucleosomes.

**Figure 2.**
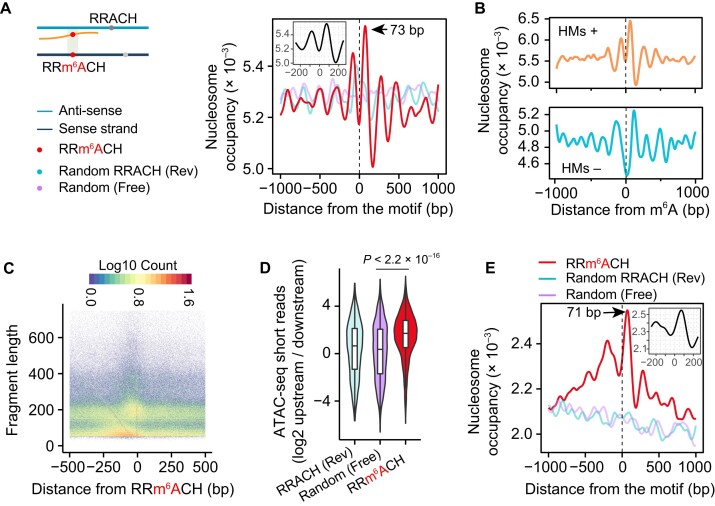
Nucleosome occupancy around m^6^A sites. (**A**) The left panel shows a schematic view of the m^6^A sites and unmodified motifs used as negative controls. The right panel shows nucleosome occupancy around m^6^A sites and unmodified motifs. The lines are smoothed by the R function smooth.spline with df = 30. A line plot at the top left shows nucleosome occupancy close to m^6^A sites (−200 bp to +200 bp). (**B**) Nucleosome occupancy around the m^6^A sites overlapping (top panel, HMs+) or not overlapping (bottom panel, HMs −) with histone modifications. (**C**) A heatmap showing the count of ATAC-seq fragments with different lengths around m^6^A sites. The 5′ end of the fragments was counted. (**D**) A violin plot showing the ratio of the short reads in the regions before m^6^A sites over the regions after m^6^A sites (Wilcox-test, *P* < 2.2 × 10^–16^). (**E**) An aggregation plot showing nucleosome occupancy around m^6^A sites or unmodified motifs. The lines are smoothed by the R function smooth.spline with df = 30. A line plot at the top right showing nucleosome occupancy close to m^6^A sites (−200 bp to +200 bp).

To explore this possibility, we took advantage of MNase-seq data in MEF cells to predict nucleosome positions and calculate nucleosome occupancy around m^6^A sites. As a negative control, we randomly generated positions on the sense strand of DNA for each m^6^A site (Random Free, Figure 2A). In addition, to exclude any potential bias in nucleosome occupancy caused by the RRACH motif (43), we randomly selected an RRACH motif on the reverse strand of DNA (Random Rev). When aligned to m^6^A sites, we found that nucleosome occupancies show a clear periodicity of approximately 200 bp, which is absent from unmethylated motifs. m^6^A sites were enriched in the nucleosome-free region flanked by two single nucleosomes, with the first downstream nucleosome placed ∼75 bp from the m^6^A site. We repeated our analysis in mouse embryonic stem cells (ESCs), which again revealed an obvious nucleosome occupancy peak in the downstream region of m^6^A sites (Supplementary Figure S3A). The association between m^6^A and nucleosome occupancy also holds true when separating m^6^A sites into introns and exons (Supplementary Figure S3B). Notably, the observed association is not affected by histone modifications, as m^6^A sites not overlapping with histone modifications also exhibited an obvious enrichment of downstream nucleosomes (Figure 2B).

We further validated the results by re-analyzing the assay of transposase accessible chromatin sequencing (ATAC-seq) datasets in MEF cells (25), which can determine both chromatin accessibility and nucleosome positions across the genome. The fragment length of ATAC-seq displayed two distinct peaks (Figure 2C and Supplementary Figure S3C): short fragments (<100 bp) and long fragments (>140 bp). The long fragments indicate DNA regions protected by nucleosomes, whereas short fragments are mainly derived from nucleosome free regions (44). In support of the association between m^6^A and nucleosome occupancy, we observed a clear enrichment of long fragments around the m^6^A sites (Figure 2C) and short fragments before m^6^A sites (Figure 2D). Importantly, the pattern disappeared when unmethylated motifs were considered (Supplementary Figure S3D). By identifying nucleosome positions using ATAC-seq long fragments (>140 bp), our analysis again uncovered a distinct peak of nucleosome occupancy approximately 75 bp downstream of m^6^A sites (Figure 2E).

In summary, our analysis in MEF cells and ESCs uncovered an association, which is unappreciated by previous studies, between m^6^A sites and the downstream nucleosomes. This association was observed irrespective of the specific histone modifications, suggesting potential curial roles of nucleosomes, independent to histone modifications, in m^6^A methylation.

### Transcription elongation rate is linked to m^6^A sites in nearby regions

The association between m^6^A and downstream nucleosomes could suggest that m^6^A plays a role in facilitating nucleosome reformation during transcription, thereby regulating nearby chromatin accessibility. However, chromatin accessibility near m^6^A sites was barely changed by the knockdown of the m^6^A methyltransferase METTL3 (Supplementary Figure S3E), suggesting that m^6^A methylation is not the reason for increased downstream nucleosome occupancy.

**Figure 3.**
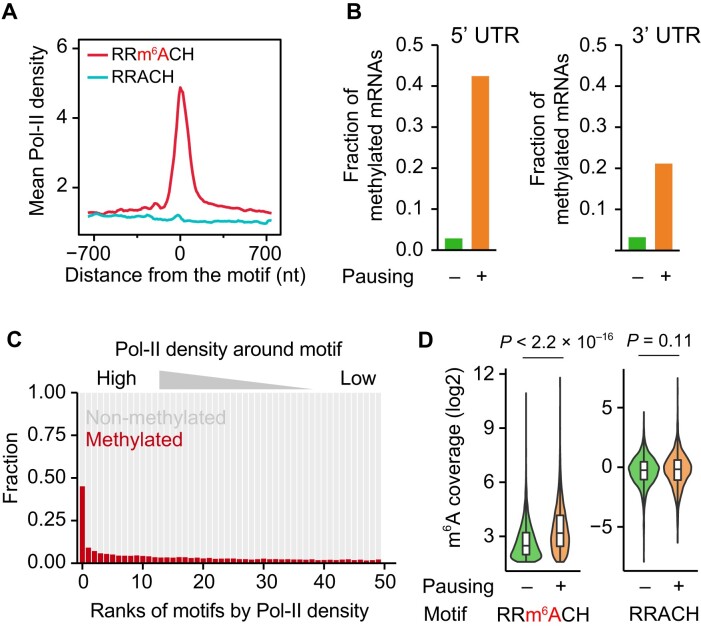
Association of m^6^A methylation with Pol-II movement. (**A**) An aggregation plot showing the average Pol-II density around m^6^A sites or RRACU motifs without modification. (**B**) Fraction of mRNAs with m^6^A in the 5′ UTR (left) or 3′ UTR (right). Genes with top (pausing) and bottom (non-pausing) 5% Pol-II densities at the early stage of elongation (left) or during transcription termination (right) were compared. (**C**) Fraction of methylated motifs. For each gene, RRACH motifs within the gene were ranked by Pol-II density. ‘0’ indicates the motifs with the highest Pol-II density among all RRACH motifs within a gene. The Y-axis shows the fraction of methylated motifs among the motifs with the same rank. Only the top 50 motifs are shown. (**D**) Comparison of m^6^A coverage (normalized by RNA-seq) between pausing and non-pausing m^6^A. The unmodified motif RRACH was used as a negative control.

During transcription, nucleosomes can act as a speed bump to regulate the movement of RNA polymerase (45,46), affecting various co-transcriptional processes (47–49). Analogous to co-transcriptional splicing (49), the enrichment of downstream nucleosomes prompted us to explore whether nucleosome-mediated transcriptional dynamics play a role in m^6^A methylation and the regional preference. While previous studies have reported that the overall m^6^A methylation level is affected by either transcription initiation and elongation rate (23,50), the transcripts and specific regions of transcripts, if any, that are sensitive to transcription rate remain undetermined. To this end, we analyzed precision run-on sequencing (PRO-seq) data in MEF cells (26). PRO-seq offers single nucleotide resolution of the 3′ end of nascent RNA, revealing the positions of engaged and active RNA polymerase across the genome. Given that most m^6^A sites are found on protein-coding RNAs, we thus focused our analysis on RNA polymerase II (Pol-II). Intriguingly, our analysis revealed a remarkable increase in Pol-II density around m^6^A sites (Figure 3A), suggesting a slowdown of Pol-II movement around m^6^A sites. The association between Pol-II density and m^6^A sites was maintained in both exons and introns (Supplementary Figure S4A). In addition, we validated this association using NET-seq and a single resolution m^6^A dataset in human HeLa cells, which again exhibited obvious Pol-II accumulation around m^6^A sites (Supplementary Figure S4B), suggesting that the observed association is conserved across species.

Pol-II pausing is found at different stages of transcription (51), especially during the early stage of elongation and transcription termination corresponding to the 5′ UTR and 5′ UTR of mRNAs. We thus investigated whether the observed association holds true in the local regions. Notably, we found that > 40% of genes with Pol-II pausing at the early stage of elongation exhibited at least one m^6^A site in the 5′ UTR of the mRNA (Figure 3B). In contrast, the fraction was reduced to 5% for the genes without Pol-II pausing at the early stage of elongation. A similar enrichment of m^6^A methylation was observed in the 3′ UTR of the mRNAs in the presence of Pol-II pausing during transcription termination. Considering all RRACH motifs on mRNAs, ∼45% of motifs with the highest Pol-II density in a transcript were methylated (Figure 3C). In contrast, the fraction of methylated motifs was less than 10% for the motifs with lower Pol-II density. Furthermore, the methylated motifs with Pol-II pausing showed significantly higher methylation levels than those without Pol-II pausing (Figure 3D, Wilcox-test, *P* < 2.2 × 10^–16^).

The strong association between m^6^A sites and Pol-II density could imply that m^6^A slows Pol-II movement. To test this possibility, we analyzed Pol-II densities around m^6^A sites in MEF cells with and without METTL3 knockdown. Notably, Pol-II densities around the vast majority of m^6^A sites (>97.5%) were barely changed by METTL3 knockdown (Supplementary Figure S4C), arguing against the possibility that m^6^A leads to Pol-II accumulation. In contrast, the number of m^6^A sites with significantly increased Pol-II densities upon METTL3 knockdown was even higher than that of m^6^A sites with decreased Pol-II density (262 sites vs.146 sites, Supplementary Figure S4D). Taken together, using single-nucleotide resolution assays for transcription rate, our analysis demonstrated a dramatically reduced elongation rate around m^6^A sites.

### Inhibiting Pol-II movement increases m^6^A methylation in nearby regions

The association between m^6^A and Pol-II movement prompted us to investigate the possibility that Pol-II movement mediates m^6^A methylation levels. Supporting this assumption, we found that actinomycin D (ActD), a transcription elongation inhibitor, increases m^6^A methylation on chrRNAs in wild-type ESCs, but not in ESCs lacking METTL3 (Supplementary Figure S5A). In contrast, blocking transcription initiation by triptolide, an initiation inhibitor that inhibits the formation of transcription bubbles and hence the initiation of transcription (52), has minimal impact on m^6^A methylation.

**Figure 4.**
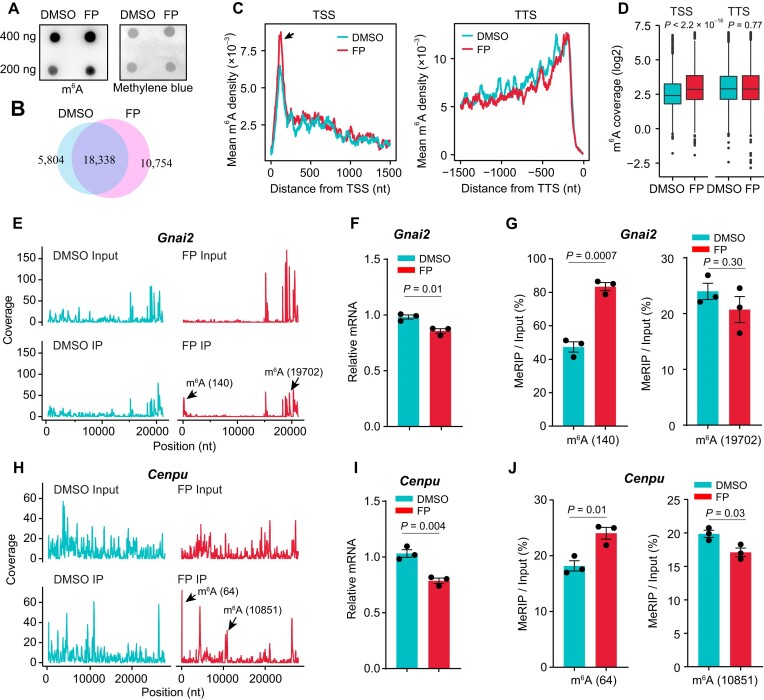
Inhibiting Pol-II movement increases m^6^A methylation. (**A**) m^6^A dot plot showing the m^6^A methylation level in MEF cells before and after flavopiridol (FP) treatment. (**B**) A Venn diagram showing m^6^A sites identified by m^6^A-seq of chrRNAs in MEF cells before and after flavopiridol (FP) treatment. (**C**) Line plots showing m^6^A densities around the transcription start site (TSS, left) and transcription termination site (TTS, right). FP: flavopiridol. (**D**) Boxplots showing m^6^A coverage, normalized by corresponding RNA levels, around the transcription start site (TSS, left) and transcription termination site (TTS, right) in MEF cells before and after flavopiridol (FP) treatment. (**E** and **H**) Line plots showing coverage of m^6^A-seq and RNA-seq reads on the genes *Gnai2* and *Cenpu*. FP: flavopiridol. (**F** and **I**) mRNA levels measured by RT-qPCR. 18S rRNA was used as an internal control. Error bars: mean ± s.e.m. FP: Flavopiridol. Two-tailed *t*-test was used to calculate the significance. (**G** and **J**) m^6^A methylation levels measured by RT-qPCR normalized by corresponding mRNA levels. Two primers based on the positions of m^6^A sites were used. m^6^A (140) and m^6^A (19702) refer to m^6^A positions at 140 nt and 19702 nt on the gene *Gnai2*. m^6^A (64) and m^6^A (10851) refer to m^6^A positions at 64 nt and 10851 nt on *Cenpu*. Error bars: mean ± s.e.m. FP: flavopiridol. Two-tailed *t*-test was used to calculate the significance

To further investigate the role of the transcription elongation rate in m^6^A methylation, we conducted chrRNA m^6^A-seq in the cells treated with a transcription elongation inhibitor. We noted that ActD and many other inhibitors such as Camptothecin used in previous studies (23) could lead to DNA breakages (53,54). Moreover, the pausing sites induced by those inhibitors are non-specific, introducing potential confounding factors in establishing the association between transcription elongation rate and specific m^6^A sites. We therefore opted for another transcription elongation inhibitor flavopiridol, which blocks P-TEFb kinase activity, thus preventing promoter-proximal pausing release (55). Flavopiridol enhances promoter-proximal pausing, specifically arresting Pol-II in the downstream region close to the TSS. Therefore, this inhibitor enables us to investigate m^6^A methylation in a specific region. When MEF cells were treated with 300 nM flavopiridol for 30 min, we observed a dramatic reduction in protein-coding mRNA production, compared to other non-coding RNAs produced by Pol-III (Supplementary Figure S5B). Intriguingly, despite the reduction in mRNA levels, the m^6^A dot plot showed comparable m^6^A levels before and after flavopiridol treatment (Figure 4A). We subsequently performed m^6^A-seq of chrRNAs in MEF cells with or without flavopiridol treatment. Consistent with the m^6^A dot plot, our m^6^A-seq data showed an increase in m^6^A sites upon flavopiridol treatment (Figure 4B). Interestingly, the m^6^A sites in the downstream region close to TSS were significantly increased (Figure 4C and D, Wilcox-test, *P* < 2.2 × 10^–16^), consistent with Pol-II pausing positions induced by flavopiridol. In contrast, m^6^A sites in downstream regions far from the TSS remained similar before and after flavopiridol treatment.

We further validated the transcriptome-wide analysis by examining m^6^A on individual genes *Gnai2* and *Cenpu*, which displayed a significant decrease in mRNA levels after flavopiridol treatment (Figure 4E, F and H, I, *t*-test, *P* < 0.05). Notably, we observed a significant increase in m^6^A levels in the downstream region close to the TSS (m^6^A 140 nt for *Gnai2* and m^6^A 64 nt for *Cenpu*, Figure 4G and J). As a negative control, m^6^A levels within the gene body remained unchanged (*Gani2*, Figure 4G) or were significantly reduced (*Cenpu*, Figure 4J). Taken together, our data suggest that inhibiting Pol-II movement increases nearby m^6^A methylation levels.

### RNA secondary structures prevent local m^6^A methylation

m^6^A is deposited onto nascent RNAs in a co-transcriptional manner. The selection of methylation sites can be affected by various factors, including RNA sequence characteristics, RNA/DNA binding proteins, and transcription factors. Sequence features impact both the selection of methylation sites and the levels of methylation (11,56). In addition to the well-documented RRACH motif, m^6^A methylation efficiency appears to be modulated by RNA structures surrounding the motif (57).

**Figure 5.**
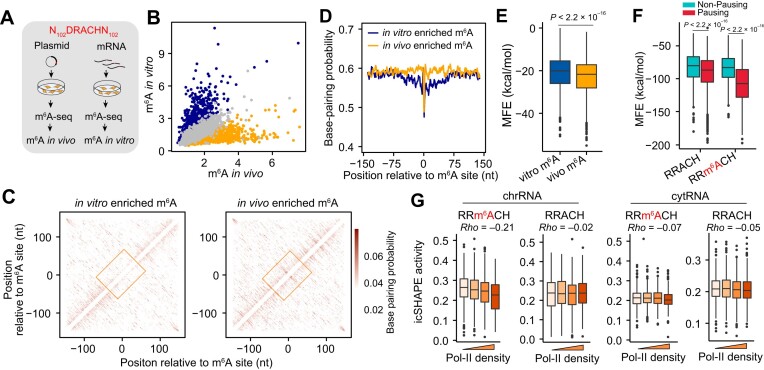
Effect of RNA secondary structures on m^6^A methylation. (**A**) A schematic diagram showing the massively parallel assays for m^6^A. m^6^A motifs with surrounding sequences (102 nt at each site) were inserted into plasmids or corresponding mRNAs. The plasmids or mRNAs were then transfected into cells, and m^6^A methylation of transfected motifs was measured by m^6^A-seq. For mRNA transfection, a recombinant METTL3-METTL14 was overexpressed in the cytosol. (**B**) A scatter plot showing correlation between *in vivo* m^6^A and *in vitro* m^6^A methylation. Differential analysis was performed using DEseq2 to determine *in vivo* enriched m^6^A motifs (orange, FDR < 0.05) and *in vitro* m^6^A enriched motifs (dark blue, FDR < 0.05). (**C**) A heatmap showing the base pairing probability of the sequences around *in vitro* enriched m^6^A motifs (left) and *in vivo* enriched m^6^A motifs (right). The x-axis shows the first position of a base pair on the mRNA, and the y-axis shows the second position of the pair (mate) on mRNA. The color indicates the probability of the base pair. (**D**) Base pair probability around m^6^A sites. The base pair probability of a position (y-axis) was calculated as the total probability of that position pairing with any other positions. (**E**) A boxplot showing minimum folding free energy (MFE) of the sequences around *in vitro* enriched m^6^A motifs and *in vivo* enriched m^6^A motifs. A lower MFE value indicates a more stable secondary structure. (**F**) A boxplot showing minimum folding free energy (MFE) around the m^6^A sites or unmethylated motifs with or without Pol-II pausing. (**G**) A boxplot showing the correlation of icSHAPE activity of cytosolic RNAs (cytRNAs) to Pol-II density around chr-m^6^A sites (left panel) or unmodified RRACH motifs (right panel). A higher icSHAPE activity indicates a lower base pairing probability.

Our previous study revealed an increase in *in vitro* RNA secondary structures around m^6^A sites (58), implying that RNA secondary structure could affects m^6^A methylation. To test this possibility, we took advantage of datasets of massively parallel assays for m^6^A (17), which provide parallel measurements of m^6^A methylation levels *in vivo* (plasmid-based) and *in vitro* (mRNA-based). In particular, *in vivo* m^6^A methylation was obtained by transfecting plasmids inserting m^6^A candidate motifs and surrounding sequences (±120 nt), where m^6^A is expected to be deposited during transcription (Figure 5A). On the other hand, *in vitro* m^6^A methylation was obtained by transfecting corresponding mRNAs containing m^6^A candidate motifs into cells that overexpress the methyltransferase complex in the cytosol. Therefore, *in vitro* m^6^A should be deposited independent to transcription. Interestingly, we observed a weak correlation between *in vivo* methylation and *in vitro* methylation, with a large number of m^6^A sites biased toward either *in vivo* or *in vitro* methylation (Figure 5B). To understand why some m^6^A sites are deposited *in vivo* but absent *in vitro*, we analyzed RNA folding of the sequences surrounding the m^6^A motifs using ViennaRNA software (59), which predicts the probability of nucleotides that are paired and the minimum folding free energy of the RNA secondary structure based on sequence information. As a result, *in vivo* enriched m^6^A sites have a higher probability pairing with surrounding nucleotides (Figure 5C and D), as well as lower folding free energy (indicating a more stable secondary structure, Figure 5E), than *in vitro* enriched m^6^A sites. These results suggest that m^6^A motifs embedded within RNA secondary structures tend to be methylated co-transcriptionally (more *in vivo* methylation). After transcription, the folded RNA secondary structures prevent these motifs from methylation (less *in vitro* methylation).

Since we have found that Pol-II pausing increases the m^6^A methylation level, and previous studies have revealed cross-talk between Pol-II movement and RNA folding (60,61), it is possible that the slowdown of Pol-II movement around m^6^A sites affects RNA folding, subsequently influencing m^6^A methylation. To explore this possibility, we extracted m^6^A sites with Pol-II pausing (pausing m^6^A, m^6^A with top 10% Pol II density) and without pausing (non-pausing m^6^A, m^6^A with bottom 10% Pol II density). We first compared the structural potential of RNA regions around m^6^A sites using ViennaRNA software. Notably, m^6^A sites with Pol-II pausing showed significantly increased structural potential compared with non-pausing m^6^A sites or motifs without modification (Figure 5F, Wilcox-test, *P* < 2.2 × 10^–16^). We further confirmed this association using *in vivo* RNA folding datasets (32), which employed icSHAPE-seq to detect *in vivo* RNA secondary. icSHAPE-seq (62) uses a chemical NAI-N3 to label unpaired nucleotides followed by selective enrichment of NAI-N3 modified RNA, thus higher icSHAPE activities indicate a lower probability of base pairing, and *vice versa*. In line with our *in silico* prediction, Pol-II densities around m^6^A sites negatively correlated with icSHAPE activities of chrRNAs (Figure 5G). By contrast, neither cytoplasmic RNAs nor non-methylated RRACH in chrRNAs exhibited a significant correlation (*P* values > 0.05) between Pol-II densities and icSHAPE activities (Figure 5G). These results suggest a Pol-II rate-dependent regulation of RNA folding around m^6^A sites.

In summary, our data suggest model that RNA secondary structures inhibit m^6^A methylation, while paused Pol-II can mediate the surrounding RNA secondary structures, increasing the accessibility of m^6^A motifs to the methyltransferase complex and consequently elevating m^6^A methylation levels.

## Discussion

Previous studies have revealed widespread roles of m^6^A methylation in gene expression. m^6^A orchestrates local biological processes through interactions with a variety of binding proteins. For instance, m^6^A recruits the low-complexity protein hnRNPG to splicing sites, affecting RNA co-transcriptional splicing (63). Furthermore, m^6^A located near TSS sites has been shown to mediate the release of TSS-promoter proximal pausing of RNA polymerase (15). These results highlight the crucial role of m^6^A regional preference. A fundamental question is how m^6^A regional preference is established within the transcript. MTC is known to favor m^6^A deposition in the consensus motif RRACH. However, given that RRACH motifs are abundant in transcripts and only ∼1% of the motifs are methylated, other mechanisms beyond the motif likely guide methylation site selection. Recent studies have proposed an EJC-suppression model to explain the landscape of m^6^A sites along transcripts. However, the selection of the m^6^A site within the EJC free regions remains unanswered.

There is an intricate interplay between histone modifications and m^6^A methylation (64). In this study, using the datasets in MEF cells, we unexpectedly observed a notable enrichment of nucleosomes in the downstream region of m^6^A sites. Intriguingly, this association is independent to histone modifications. This result suggests that, beyond histone modifications, the nucleosome itself may affect m^6^A methylation. Although it is plausible that nucleosomes or histones may directly recruit MTC to local regions of transcripts, we deferred this determination to future studies and shifted our focus to transcription dynamics mediated by nucleosomes. Transcription is a highly dynamic process regulated by a multitude of factors that dictate the tempo of transcription elongation. Specially, nucleosomes downstream of the Pol-II can act as physical barriers to slowdown Pol-II movement (65). Interestingly, the transcription elongation rate mirrors the distribution of m^6^A along transcripts, displaying an obvious slowdown of Pol-II movement at the early stage of elongation and during termination. Therefore, nucleosome-mediated transcription dynamics likely offer a unified explanation for the observed association between m^6^A sites, nucleosomes and histone modifications. Indeed, previous studies have hinted at a potential role of transcription rate in m^6^A methylation. For instance, the promoter-proximal pausing increases the recruitment of MTC to the TSS of active transcribed genes (15,50). Interestingly, the m^6^A methylation status of transcript associates with the promoter-proximal pausing (15,50). It remains an open issue why the promoter-proximal pausing is required for the downstream m^6^A methylation far from the TSS. In this study, using single-nucleotide resolution assay for transcription elongation, we firstly revealed that the slowdown of Pol-II increases m^6^A methylation levels in the nearby region, indicating that, in addition to the EJC-suppression model, the transcription elongation rate contributes an additional layer to drive m^6^A regional preference. Although it remains unclear whether a unified mechanism integrates these two models, one possibility is that the location of EJC determines mRNA regions open to the methyltransferase complex, while the transcription elongation rate affects the selection of m^6^A motifs (and thus methylation site) and the subsequent methylation levels.

How does the transcription elongation rate mediate m^6^A methylation levels? Interestingly, a recent study found differential m^6^A methylation levels when the m^6^A motif GGACU was placed at different positions within an RNA secondary structure (57). In addition, our previous study (58) also revealed that most METTL3-sensitive m^6^A sites are embedded within RNA secondary structures, implicating the role of RNA secondary structures in m^6^A site selection. Indeed, our analysis, employing massively parallel assays, demonstrates that m^6^A motifs shielded by RNA secondary structures can undergo methylation during transcription but are less likely to be methylated after transcription. mRNAs tend to form more secondary structures once exported from the nucleus to the cytosol. The increased secondary structures encompass m^6^A motifs, diminishing their accessibility to the methyltransferase complex. During transcription, the presence of a paused Pol-II could inhibit the folding of surrounding RNA sequences, thus increasing the likelihood of m^6^A methylation near pausing sites. Notably, after transcription, many RNA regions can refold into secondary structures unless bound by other proteins. In our previous study (58), we found m^6^A in RNA secondary structure is required for the efficient translation in RNA structure regions. Therefore, this study alongside with our previous study illustrates how m^6^A is deposited to specific regions and how the locally deposited m^6^A sites in-turn regulate biological processes in nearby regions.

In summary, we here reported that the slowdown of Pol-II movement promotes m^6^A methylation in the nearby region, contributing to m^6^A regional preference. We hypothesized that Pol-II movement affects nearby m^6^A methylation levels by influencing the accessibility of m^6^A candidate motifs to MTC via mediating RNA secondary structures. Although we have demonstrated in this study that RNA secondary structures *in vitro* inhibit m^6^A methylation and that RNA folding *in vivo* is affected by Pol-II movement, direct evidence from single-molecule assays (66,67) showing the dynamics of RNA secondary structure around Pol-II and subsequent m^6^A methylation will be critical for understanding the molecular mechanism for m^6^A site selection.

## Supplementary Material

gkae169_Supplemental_File

## Data Availability

All sequencing data have been deposited in the National Center for Biotechnology Information Gene Expression Omnibus (GEO) and are accessible through the GEO Series accession number GSE247090.
