# Supplementary material for: Determinant of m6A regional preference by transcriptional dynamics
Source: Nucleic Acids Res. 2024 Mar 7;52(7):3510–21. doi: 10.1093/nar/gkae169 (PMC11039984; doi:10.1093/nar/gkae169)
Supplement: gkae169_Supplemental_File [file gkae169_supplemental_file.pdf]

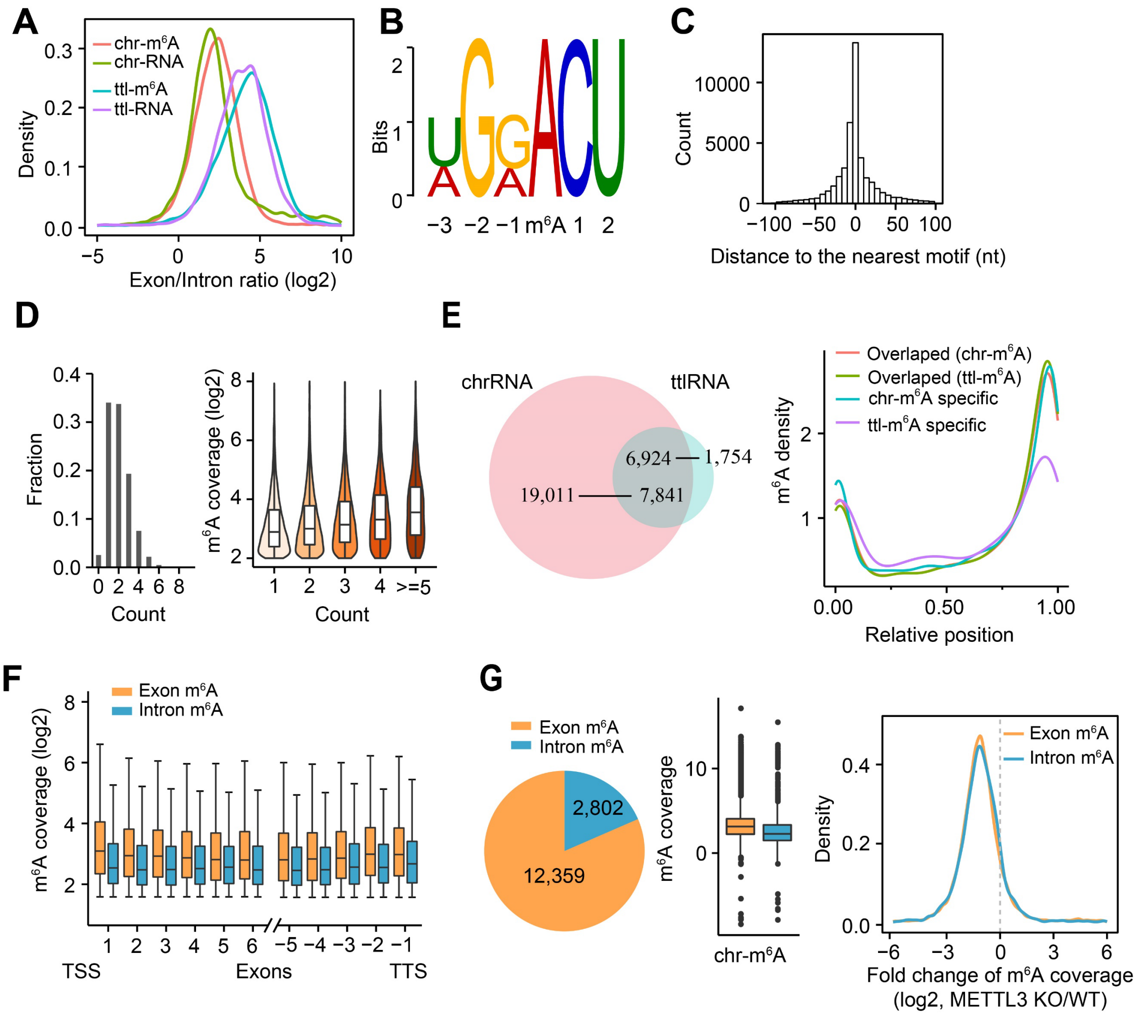

Figure S1. Quality of chrRNA m^{6}A-seq.

(A) Comparison of exon/intron ratios between chrRNAs and ttlRNAs.

(B) Consensus motif around m^{6}A peaks.

(C) The distance of identified m^{6}A coverage summit to the nearest m^{6}A motif.

(D) Left panel shows the distribution of the number of m^{6}A motifs within m^{6}A peaks. Right panel shows a positive correlation between m^{6}A coverage (normalized by RNA-seq) and the number of motifs within the m^{6}A peak.

(E) Overlapping m^{6}A sites between chrRNA and ttlRNA.

(F) Comparison of exon and intron m^{6}A sites that are assigned to different exons or introns. Exons are ranked by the distance to transcription start site (TSS, left) or transcript terminate site (TTS, right).

(G) The pie chart is the number of exon and intron m^{6}A sites in ESCs. The boxplot shows m^{6}A coverage (normalized by RNA-seq) of intron and exon m^{6}A sites. Right

panel shows the fold change of m^6A coverage (normalized by RNA-seq) in response to METTL3 knockout.

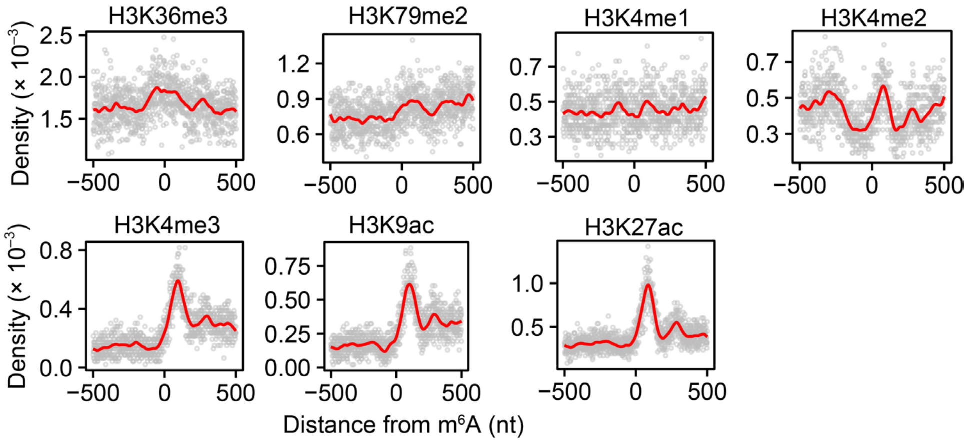

Figure S2. Analysis of histone modifications around chrRNA m^{6}A. Density of histone markers around m^{6}A sites in MEF cells (grey circle). Red lines are the smoothed density, using the R function smooth.spline() with df = 30.

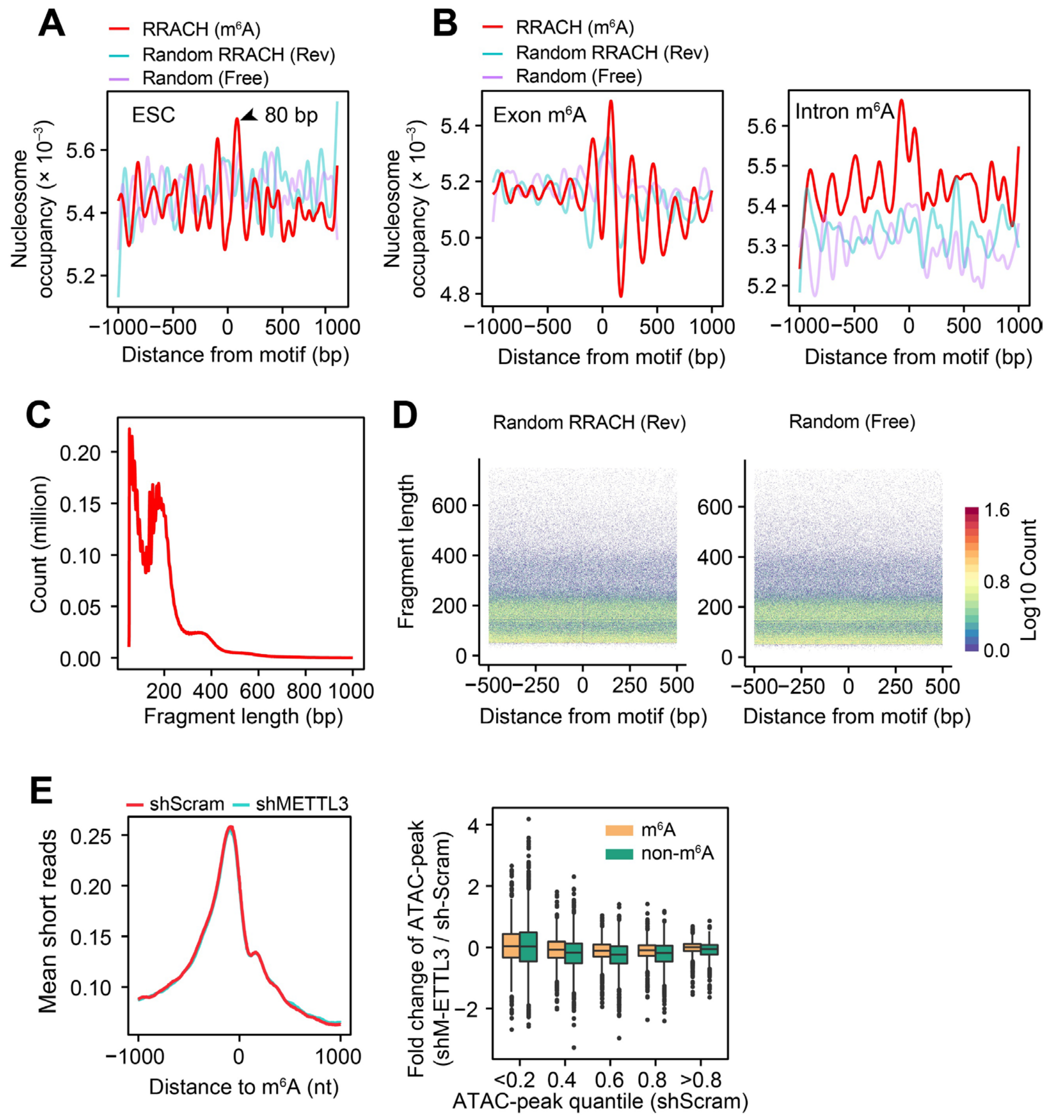

Figure S3. Analysis of nucleosome occupancy around m^{6}A sites.

(A) Nucleosome occupancy around m^{6}A sites and unmodified motifs in ESCs. The lines are smoothed by R function smooth.spline() with df =30.

(B) Nucleosome occupancy around exon (left) or intron (right) m^{6}A sites in MEF cells.

(C) Distribution of the inserted length of ATAC-seq.

(D) Heatmaps show the count of ATAC-seq reads with different inserted length around unmethylated RRACH motifs.

(E) The left line plot shows chromatin accessibility around m^{6}A sites. The right boxplot shows fold changes of the ATAC-seq peaks overlapping or not overlapping with m^{6}A sites.

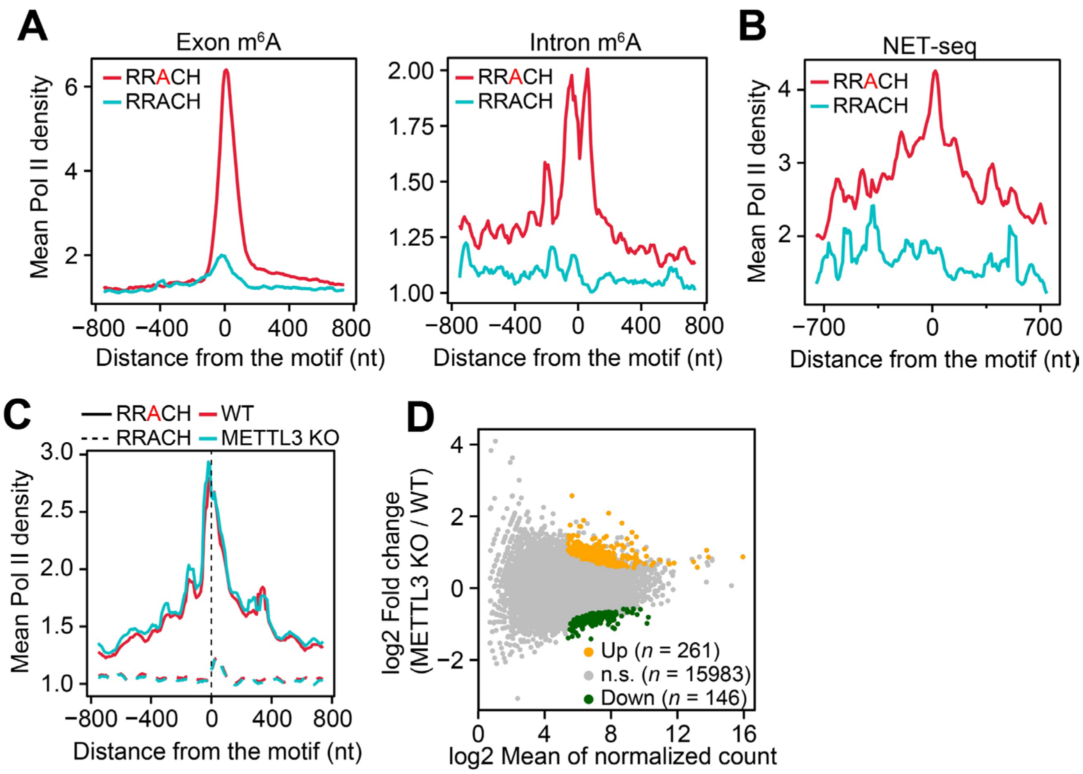

Figure S4. Association of m⁶A to transcription elongation rate.

(A) Pol-II density around exon (left) or intron (right) m^{6}A sites.

(B) Pol-II density around m^{6}A sites. Pol-II density was calculated based on mNET-seq in HeLa cells. Single-nucleotide m^{6}A sites on chromatin associated mRNAs in HeLa cell were used.

(C) Pol-II density around m^{6}A sites or unmethylated motifs in the cells with or without METTL3 depletion.

(D) A MA-plot shows the m^{6}A sites with significant increase (Up) or decrease (Down) Pol-II densities upon METTL3 knockout.

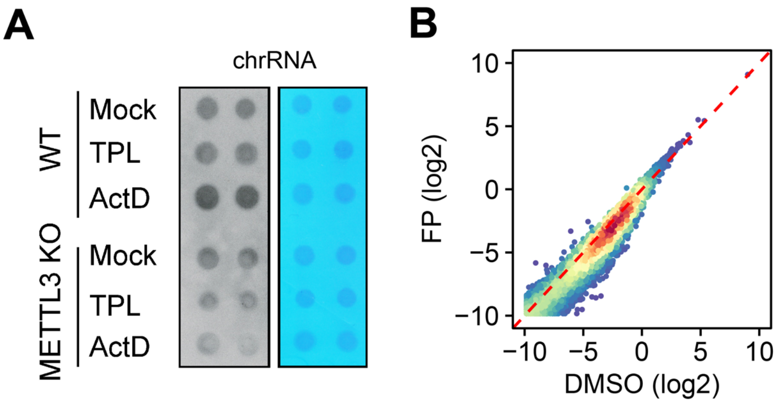

Figure S5. Effect of transcription inhibition on chrRNA expression and m^{6}A.

(A) m^{6}A methylation levels of the RNAs in different cellular fractions. TPL: Triptolide, ActD: Actinomycin D. chrRNA: chromatin associated RNAs.

(B) A scatter plot showing expression levels of chromatin associated mRNAs before and after Flavopiridol treatment. FP: Flavopiridol. mRNA levels were normalized by the level of snoRNAs.
